# Malaria research in Malawi from 1984 to 2016: a literature review and bibliometric analysis

**DOI:** 10.1186/s12936-017-1895-8

**Published:** 2017-06-12

**Authors:** Chikondi A. Mwendera, Christiaan de Jager, Herbert Longwe, Charles Hongoro, Clifford M. Mutero, Kamija S. Phiri

**Affiliations:** 1 University of Pretoria Institute for Sustainable Malaria Control (UP ISMC), School of Health Systems and Public Health, University of Pretoria, Private Bag X363, Pretoria, 0001 South Africa; 2 ICAP at Columbia University, Mailman School of Public Health, Pretoria, South Africa; 3 Population Health, Health Systems and Innovation, Human Sciences Research Council (HSRC), Pretoria, South Africa; 4 International Centre of Insect Physiology and Ecology (ICIPE), P.O. Box 30772, Nairobi, Kenya; 5 School of Public Health and Family Medicine, College of Medicine, University of Malawi, Blantyre, Malawi

**Keywords:** Malaria research, Funding, Health policy, Malawi

## Abstract

**Background:**

Malaria research can play a vital role in addressing the malaria burden in Malawi. An organized approach in addressing malaria in Malawi started in 1984 by the establishment of the first National Malaria Control Programme and research was recognized to be significant. This study aimed to assess the type and amount of malaria research conducted in Malawi from 1984 to 2016 and its related source of funding.

**Methods:**

A systematic literature search was conducted in the Medline/PubMed database for Malawian publications and approved malaria studies from two Ethical Committees were examined. Bibliometric analysis was utilized to capture the affiliations of first and senior/last authors, funding acknowledgements, while titles, abstracts and accessed full text were examined for research type.

**Results:**

A total of 483 publications and 165 approved studies were analysed. Clinical and basic research in the fields of malaria in pregnancy 105 (21.5%), severe malaria 97 (20.1%) and vector and/or agent dynamics 69 (14.3%) dominated in the publications while morbidity 33 (20%), severe malaria 28 (17%) and Health Policy and Systems Research 24 (14.5%) dominated in the approved studies. In the publications, 146 (30%) first authors and 100 (21%) senior authors, and 88 (53.3%) principal investigators in approved studies were affiliated to Malawian-based institutions. Most researchers were affiliated to the Malawi-Liverpool Wellcome Trust, College of Medicine, Blantyre Malaria Project, Ministry of Health, and Malaria Alert Centre. The major malaria research funders were the National Institute for Health/USA, Wellcome Trust and the US Agency for International Development. Only three (2.5%) out of 118 journals publishing research on malaria in Malawi were from Africa and the *Malaria Journal*, with 76 (15.7%) publications, published most of the research from Malawi, followed by the *American Journal of Tropical Medicine and Hygiene* with 57 (11.8%) in comparison to only 13 (2.7%) published in the local *Malawi Medical Journal*.

**Conclusions:**

Clinical and basic research, which is mostly funded externally, in the fields of malaria in pregnancy, severe malaria and vector and/or agent dynamics dominated, while health policy and system research was least supported. The quantity may reflect scientific research activity but the initial primary impact is contribution to policy development.

## Background

Research is defined as an organized curiosity leading to a systematic enquiry, with the purpose of understanding the subject at hand and generating new knowledge. This definition has been applied in health research as the production of new knowledge using scientific methods to identify and tackle health problems [1]. Research can therefore play a vital role in health by understanding disease dynamics and discovering new interventions of treatment and prevention. Developed countries have made major strides and impacted enormously on the global health research arena with little contribution from developing countries. Due to contextual differences, challenges exist in the generalization and applicability of health research findings to different settings, hence locally available evidence is critical [2]. Developing countries should also realize that only 5% of funding for global health research is devoted to address their research needs where 90% of health problems exist [3]. Health research has been identified to be critical in providing evidence for decision-making, leading to development of interventions addressing health problems in the world [2]. All efforts should thus be made for such research evidence, which is one step towards policy change [4], to be translated into policy and practice in order to attain the ultimate goal of improving public health. It is against this background that developing countries should invest and conduct robust health research and utilize it for policy development and planning to improve health systems and avert preventable health burdens [2, 5].

Malawi, as a resource-limited developing country, faces many health challenges requiring great attention. Malaria is ranked third on major disease burdens in Malawi [6] with an estimated four million cases occurring annually, mostly in pregnant women and children under 5 years old [7]. An organized approach in addressing the malaria burden in Malawi started in 1984 by the establishment of the first National Malaria Control Programme (NMCP) and the development of its national malaria control policy. One of the policy directions was to conduct viable research to guide the development of policies in malaria treatment, control and prevention [8]. This development was in line with the recommendation by the Commission on Health Research for development in developing countries in 1990 to increase capacity of health research in developing countries [1]. It is imperative to assess whether these efforts have had any impact through the health research output in malaria as recommended by the Organization for Economic Cooperation and Development (OECD) in describing research activity for a country [9].

The aim of this study was to assess the malaria research output by mapping the type and amount of malaria research conducted in Malawi since 1984 when the first NMCP was established to 2016 when this study was conducted. The assessment also describes affiliations, level of collaborations and the sources of funding for malaria research in Malawi. This assessment also forms part of a larger study promoting malaria research utilization in policy development that should eventually lead to the development of evidence-based interventions to address the malaria burden in Malawi. The research promotion will be instituted by the development of a contextual malaria research-to-policy framework. One of the initial steps in the development of this framework is to verify the availability of malaria research conducted in Malawi and create a malaria research repository.

## Methods

An online systematic literature search was conducted for published primary research from Malawi and the examination of approved malaria studies by the two Ethical Committees (ECs) in Malawi, namely the National Health Sciences Research Committee (NHSRC) and the College of Medicine Research and Ethics Committee (COMREC).

### Published literature

An online Medline/PubMed database search was conducted to capture malaria publications from Malawi since 1984–2016, with the latest search conducted on 9 January 2017. The Medline/PubMed, an online international database, was chosen as the only database searched because it freely provides access to over 5000 peer reviewed indexed journals which are periodically updated by the US National Library of Medicine and hence it is bound to capture a large number of viable research publications [10, 11]. The medical subject headings (MESH) tool was used by combining Boolean ‘AND’ of malaria and Malawi terms as follows: (‘malaria’ [MeSH Terms] OR ‘malaria’ [All Fields]) AND (‘Malawi’ [MeSH Terms] OR ‘Malawi’ [All Fields]) AND (‘1984/01/01’ [PDAT]: ‘2016/12/31’ [PDAT]).

### Inclusion and exclusion criteria

Primary malaria research conducted in Malawi was included in the review, and multi-country primary research that involved collection of primary data from Malawi. The study excluded commentaries, systematic reviews and meta-analyses, and research articles that only referenced malaria research conducted in Malawi. However, original studies from Malawi referenced and included in the systematic reviews and meta-analysis were sought and incorporated in the analysis.

### Approved malaria studies

A list of approved malaria studies from the two ECs in Malawi, NHSRC and COMREC was obtained. The assumption was that all viable health research conducted in Malawi undergoes ethical approval and its records should be accessible at these ECs. The extraction of these studies required coverage from the periods when the ECs were established. The NHSRC, under the Research Unit in the Ministry of Health, was established in 1988 and mandated to review and clear all health research conducted in Malawi. However, with growing research demand, COMREC, under the College of Medicine (COM) in the University of Malawi, was established in 1996 and mandated to facilitate the review of proposals of faculty members and students of COM and Kamuzu College of Nursing, and their affiliates which include the Malawi-Liverpool Wellcome (MLW) Trust, Blantyre Malaria Project (BMP), Malaria Alert Centre (MAC), and Centre for Reproductive Health (CRH).

### Analysis plan

Bibliometric analysis, which was limited to the quantitative indicator of research activity and extent of co-authorship, was utilized [12]. This study was purely descriptive by examining the amount, trends, institutional affiliations of first and last authors, types, and sources of funding for malaria research conducted in Malawi. In addition, various relationships of the variables were established through cross-tabulations.

**Fig. 1:**
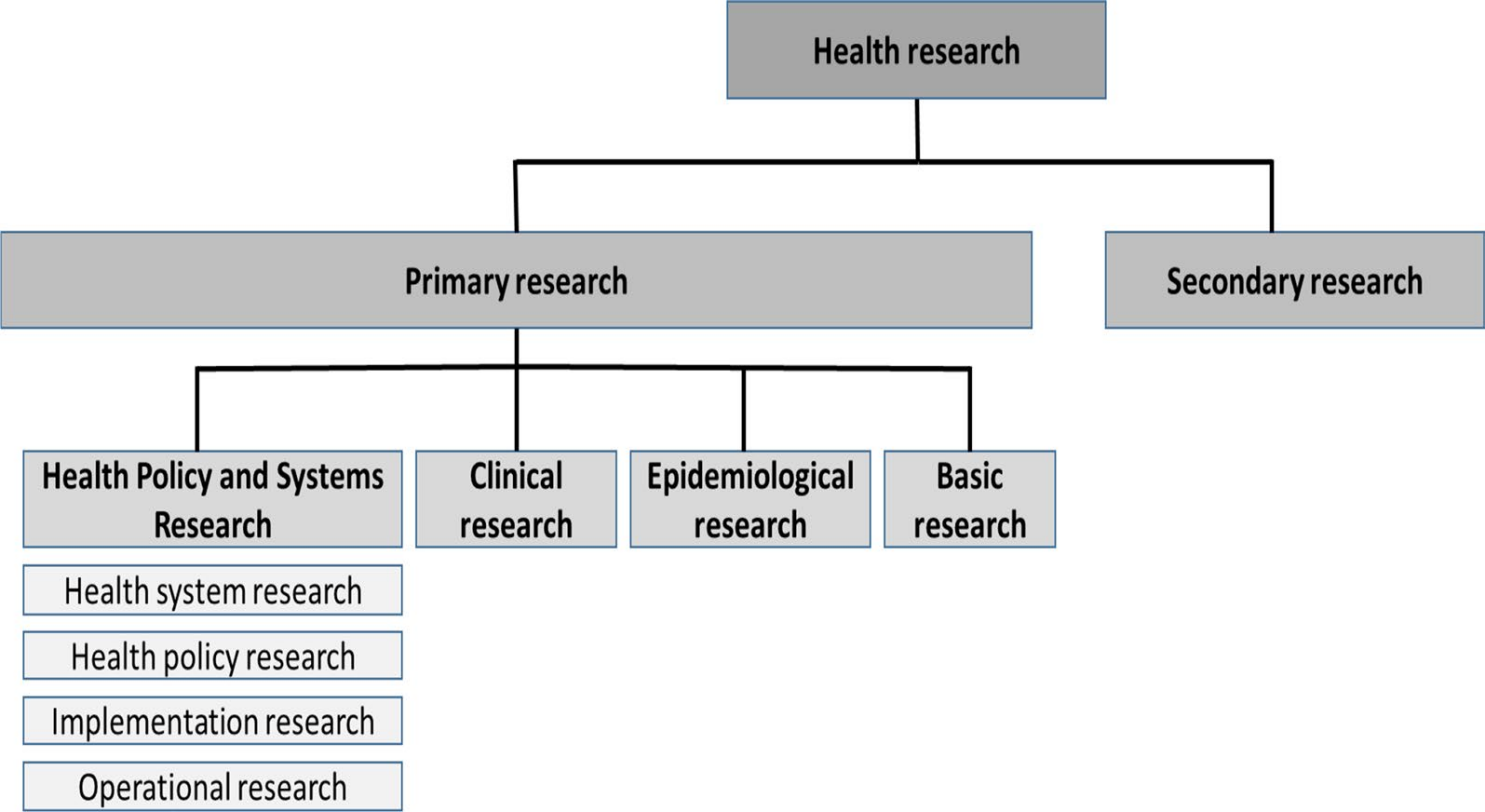
Categories of research used in the review

The analysis focused on providing outputs of the following: (1) amount of malaria research conducted in Malawi from 1984 to 2016; (2) type of malaria research studies conducted in that period; (3) institutional affiliations of first and senior/last authors in addition to local and international collaborations; and, (4) source of malaria research funding. The variables extracted from the publications and approved studies included years of publication and study approval, affiliations of the principal investigators (PIs), first and senior/last authors, and funding acknowledgements. Categorization of malaria research into various types was through inspection of the titles, abstracts and full papers, where possible. For the purposes of this review, the type of malaria research were first categorized into primary and secondary then the focus on primary research was later grouped into basic, epidemiological, clinical, and Health Policy and Systems Research (HPSR) (Table 1; Fig. 1). Analysis was further extended to areas of focus for malaria research, which included malaria in pregnancy, immunology, severe malaria, drug evaluation, morbidity, diagnosis, vector and/or agent dynamics, drug discovery, malaria vaccine, co-infections, HPSR, and prevention (research on long-lasting treated nets, indoor residual spraying, environmental sanitation, and personal protection). This categorization was done by two independent reviewers and differences were resolved on consensus and to measure the level of agreement a Cohen’s kappa score of 0.83 was calculated using the GraphPad software [13].

**Table 1:** Description of research types covered in the review

Type of research	Description of research type	Example
Secondary research	This type of research involves analysis of already conducted studies (primary research) that have been published. It involves analyzing, summarizing and interpreting relevant primary research based on the writing topic [25]	Kabaghe AN, Visser BJ, Spijker R, Phiri KS, Grobusch MP, Vugt M. Health workers’ compliance to rapid diagnostic tests (RDTs) to guide malaria treatment: a systematic review and meta-analysis. *Malaria journal*. 2016;15(1):1
Primary research	This is a type of research where the collection of primary data from subjects or experiments is involved. It is sometimes referred to as original research [26]	Includes basic, clinical, epidemiological, and Health Policy and Systems Research
Basic research	This is also referred to as fundamental or experimental research, which involves studying life processes to generate new knowledge or theories that can be applied universally. It includes among others cell studies, animal experiments, and genetic and physiological investigations [26]	Barnes KG, Irving H, Chiumia M, Mzilahowa T, Coleman M, Hemingway J, Wondji CS. Restriction to gene flow is associated with changes in the molecular basis of pyrethroid resistance in the malaria vector Anopheles funestus. Proceedings of the National Academy of Sciences. 2016;201615458
Clinical research	This can be experimental or observational with the purpose of answering specific questions on diseases and normal functioning by using human subjects. It intends to assess the safety and effectiveness of drugs, or diagnostic products for human use [26]	Dambe R, Sande J, Ali D, Chilima B, Dodoli W, Michelo C, Malenga G, Phiri KS. Monitoring the efficacy of artemether-lumefantrine for the treatment of uncomplicated malaria in Malawian children. *Malaria journal*. 2015;14(1):1
Epidemiological research	This can be descriptive, analytical or interventional with the purpose of investigating the distribution of determinants and patterns of disease frequencies in a given population. Understanding of the factors lead to strategic disease control and prevention [26]	Jonker FA, Calis JC, van Hensbroek MB, Phiri K, Geskus RB, Brabin BJ, Leenstra T. Iron status predicts malaria risk in Malawian preschool children. *PLoS One*. 2012;7(8):e42670
Health Policy and Systems Research	Its aim is to improve a health system and involves the generation of new knowledge on how societies can organize themselves for the achievements of health goals. This type of research is mainly used by policy makers and health service manager for decision making [27]	Includes Operational, implementation, health systems, and health policy research
Health systems research	This is a multidisciplinary field of scientific investigation on any or several WHO six building blocks of a health system that include service delivery, information and evidence, medical products and technology, health workforce, health financing, and leadership and governance [22]	Yoder PS, Nsabagasani X, Eckert E, Moran A, Yé Y. Perspectives of health care providers on the provision of intermittent preventive treatment in pregnancy in health facilities in Malawi. *BMC health services research*. 2015;15(1):354
Health policy research	This type of research seeks to understand the nature of health policies and the interaction of various factors in the policy development process and implementation [27]	Mwendera C, de Jager C, Longwe H, Phiri K, Hongoro C, Mutero CM. Malaria research and its influence on anti-malarial drug policy in Malawi: a case study. *Health Research Policy and Systems*. 2016;14(1):1
Implementation research	The purpose of this research is to find tangible strategies of scaling up or implementation of an existing or new intervention proven efficacious in order to improve its accessibility to the wider population [22]	Almond D, Madanitsa M, Mwapasa V, Kalilani-Phiri L, Webster J, Kuile F, Paintain L. Provider and user acceptability of intermittent screening and treatment for the control of malaria in pregnancy in Malawi. *Malaria Journal*. 2016;15(1):574
Operational research	This seeks to find solutions to address operational challenges to a specific health programme in a given area. The challenges are usually identified through the routine monitoring and evaluation activities [22]	Ewing VL, Tolhurst R, Kapinda A, Richards E, Terlouw DJ, Lalloo DG. Increasing understanding of the relationship between geographic access and gendered decision-making power for treatment-seeking for febrile children in the Chikwawa district of Malawi. *Malaria Journal*. 2016;15(1):521

The IBM Statistical Package for Social Sciences (SPSS) software version 20 was utilized for analysis, while other specific analyses and graphical outputs were also conducted in Microsoft Excel. Analysis of publications and ethically approved studies was conducted separately because studies may constitute larger studies from which specific publications may arise and that the names of the studies may not necessarily be the titles of publications.

## Results

**Fig. 2:**
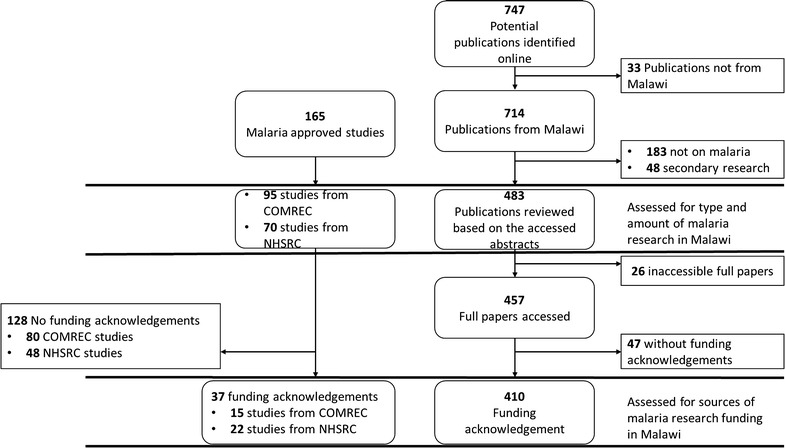
A flow chart of the selection process of studies and publication reviewed

A total of 747 potential publications were retrieved online while records of 165 approved studies were accessed from COMREC and NHSRC. After applying the inclusion and exclusion criteria to the publications, 483 publications were assessed for type and amount of malaria research from Malawi, of which 412 (85.3%) was research conducted in Malawi only and 71 (14.7%) was multi-country research which included Malawi. Furthermore, 410 publications and 37 approved studies were assessed for sources of malaria research funding (Fig. 2). However, records of approved studies in earlier years (from 1988 to 2005 from NHSRC, and from 1996 to 2005 from COMREC) were not available.

### Trend of malaria research since 1984

**Fig. 3:**
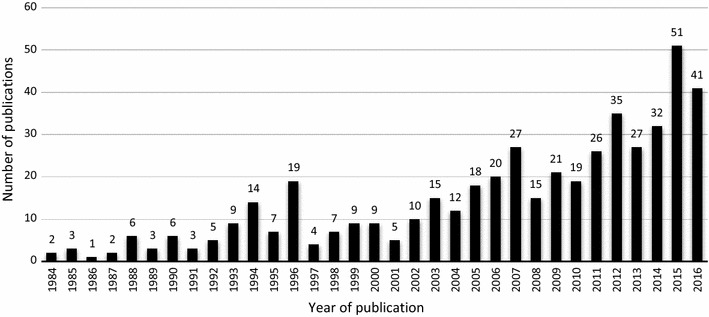
Trend of malaria publications in Malawi

The number and trend of malaria publication records for each year are presented in Fig. 3. It is evident that there has been a slow increase in the number of malaria publications from 1984 to 2001 with exceptions in 1994 and 1996 and increasing steadily from 2002 to 2016. The lowest number of publications was in 1986 with only one (0.21%) publication while the highest number was in 2015 with 51 (10.6%) publications. However, there was an average of 15 publications per year.

**Fig. 4:**
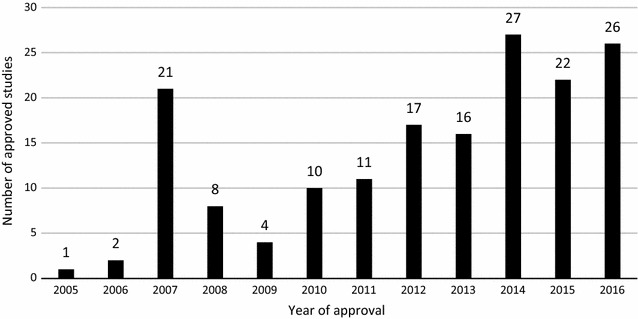
Trend of malaria approved studies in Malawi

Approved studies from ECs showed a steady increase with 2014 approving 27 studies and uniquely in 2007 when 21 studies were approved compared to the previous and later years until 2014 (Fig. 4).

### Publishing journals for malaria research in Malawi

**Fig. 5:**
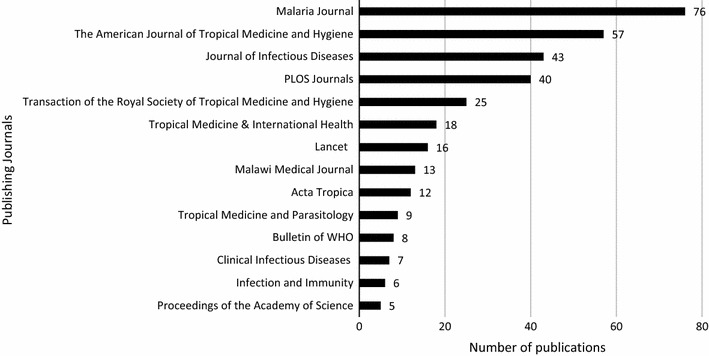
Journals publishing malaria research from Malawi

There were a total of 118 journals that published malaria research from Malawi with only three (2.5%) African journals, which included the *African Journal of Health Sciences* with one (0.2%), *African Health Sciences* with two (0.4%), and the *Malawi Medical Journal* with 13 (2.7%) publications. However, Fig. 5 shows journals with five (1%) or more publications. It shows that *Malaria Journal* registered the highest number of publications, contributing 76 (15.7%) publications, while the local *Malawi Medical Journal* contributed 13 (2.7%) publications.

### Types of research

**Fig. 6:**
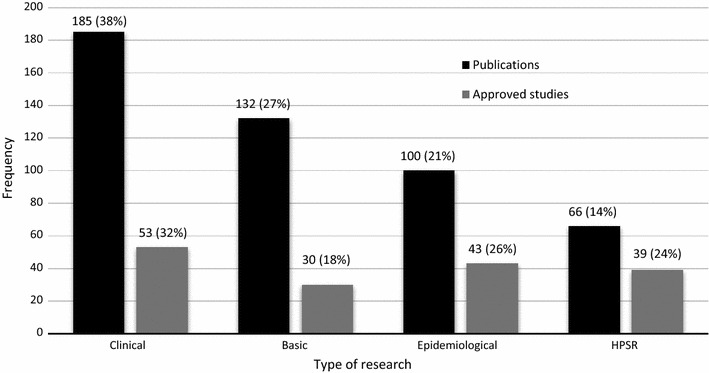
Type of malaria research conducted in Malawi

Categorizing studies into specific types posed a challenge as many studies overlapped. However, this was overcome by the agreement of two independent reviewers on disagreed studies. The first categorization of type on malaria research was based on either research being basic, clinical, epidemiological, or HPSR. Both the publications and approved studies show that clinical research was dominant with 185 (38%) and 53 (32%), respectively (Fig. 6). Further categorization of HPSR publications (n = 66), shows that 28 (43%) were health systems research, 14 (21%) were implementation research, while health policy and operation research had 12 (18%) publications each. The HPSR in the approved studies (n = 39) shows that 18 (46%) were implementation, 13 (33%) operational, six (16%) health systems, and two (5%) health policy research.

### Focus areas for malaria research conducted in Malawi

Malaria research was also assessed in relation to areas of focus. It was shown that 105 (21.7%) publications were focused in the field of malaria in pregnancy and 97 (20.1%) in severe malaria with only one publication on malaria vaccine, while morbidity studies 33 (20%), severe malaria 28 (17%) and HPSR 24 (14.5%) dominated in the approved studies (Table 2).

**Table 2:** Areas of focus for malaria research in Malawi

Field of malaria research	Number of publications	Approved studies
Malaria in pregnancy	105 (21.7%)	15 (9.1%)
Severe malaria	97 (20.1%)	28 (17%)
Vector and/or agent dynamics	69 (14.3%)	16 (9.7%)
Morbidity	64 (13.3%)	33 (20%)
Drug evaluation	52 (10.8%)	20 (12.1%)
HPSR	38 (7.9%)	24 (14.5%)
Prevention	30 (6.2%)	14 (8.5%)
Diagnosis	20 (4.1%)	6 (3.6%)
Immunology	7 (1.4%)	3 (1.8%)
Malaria vaccine	1 (0.2%)	6 (3.6%)
Total	483 (100%)	165 (100%)

Forty-two publications were also reviewed in reference to co-infections. Thirty-three (79%) out of 42 publications were on HIV and AIDS and malaria co-infection, followed by four (2%) on nutritional problems; the 12 approved studies identified were on co-infection research of malaria and HIV and AIDS.

### Affiliation of first and senior/last authors

Institutional affiliations of first and senior/last authors were assessed by examining whether they were affiliated to a Malawian or foreign institution. The results showed that 146 (30%) first authors out of 483 publications were affiliated to a Malawian institution. Forty-eight (32.8%) were affiliated to MLW followed by 31 (21.2%) at COM, University of Malawi (Table 3).

**Table 3:** Affiliations of first and senior authors in Malawi

Malawian institutions	First authors	Senior authors
Malawi-Liverpool Wellcome Trust	48 (32.8%)	30 (30%)
College of Medicine, University of Malawi	31 (21.2%)	20 (20%)
Ministry of Health	18 (12.3%)	20 (20%)
Malaria Alert Center	15 (10.3%)	8 (8%)
Chancellor College, University of Malawi	7 (4.8%)	2 (2%)
Queen Elizabeth Central Hospital	5 (3.4%)	–
Kamuzu Central Hospital	5 (3.4%)	–
Blantyre Malaria Project	5 (3.4%)	19 (19%)
St. Gabriel’s Hospital	2 (1.4%)	–
International Eye Foundation	2 (1.4%)	–
Save the Children International	2 (1.4%)	–
Centre for Water, Sanitation, Health and Appropriate Technology Department	1 (0.7%)	–
Beit Cure International Hospital	1 (0.7%)	–
Centre for Social Research	1 (0.7%)	1 (1%)
Safe Motherhood Project and Blantyre Integrated Malaria Initiative	1 (0.7%)	–
Department of Pediatrics, University of Malawi	1 (0.7%)	–
Malamulo Hospital	1 (0.7%)	–
Total	146 (100%)	100 (100%)

**Fig. 7:**
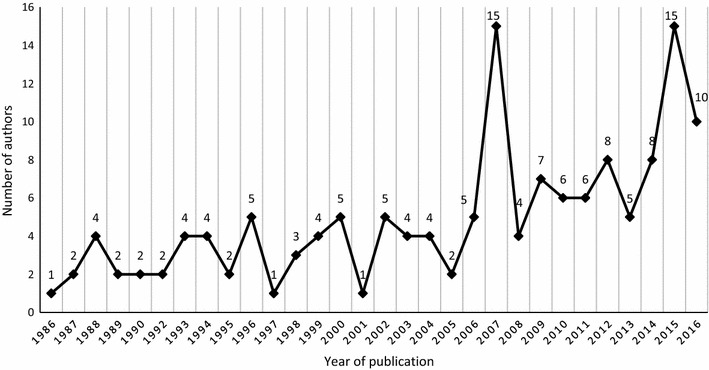
Number of first authors affiliated to Malawian Institution with time

Upon further analysis of first authors affiliated to Malawian institutions compared to year of publications, there had been a slight increase with time. The exception was observed in 2007 and 2015 with 15 publications each with first authors in Malawian institutions (Fig. 7).

Senior authorship was assessed through examination of the last author, who is considered to be the supervisor or senior member of the team [14]. The results showed that 100 (21%) senior authors out of 483 publications were affiliated to an institution based in Malawi. Thirty (30%) were affiliated to MLW followed by 20 (20%) to COM and MOH each (Table 3).

In the 165 approved studies assessed 88 (53.3%) PIs were affiliated to a Malawian institution, 16 (9.7%) to a foreign institution while the affiliation of 61 (37%) PIs was unknown as it was not indicated in the records. Table 4 shows that 20 (23%) PIs were affiliated to COM and 12 (14%) to MAC, BMP and UNC project each.

**Table 4:** Affiliations of principal investigators (PIs) in Malawi

Malawian institution	Number of PIs
College of Medicine, University of Malawi	20 (23%)
Malaria Alert Centre	12 (14%)
Blantyre Malaria Project	12 (14%)
University of North Carolina Project	12 (14%)
Malawi-Liverpool Wellcome Trust	11 (13%)
Ministry of Health	10 (11%)
Malawi College of Health Sciences	3 (3%)
UNICEF/Malawi	2 (2%)
Mzuzu University	1 (1%)
Chancellor College	1 (1%)
Deayang Luke Hospital	1 (1%)
John Hopkins research project/Malawi	1 (1%)
REACH Trust	1 (1%)
Save the Children International	1 (1%)
Total	88 (100%)

### Collaboration

Collaboration was ascertained when there was an affiliation between a Malawian and a foreign institution as indicated in the authors’ affiliations. Out of 483 publications, 350 (72%) showed a collaboration, while 120 (25%) indicated foreign institutions only and 13 (3%) were Malawian institutions only.

Collaboration in the approved studies was not established because only the institution of the PI was indicated in the records. However, it is a requirement that foreign institutions conducting research in the country should be affiliated to a local institution and incorporate local researchers for purposes of collaboration and capacity building.

### Sources of malaria research funding in Malawi

**Fig. 8:**
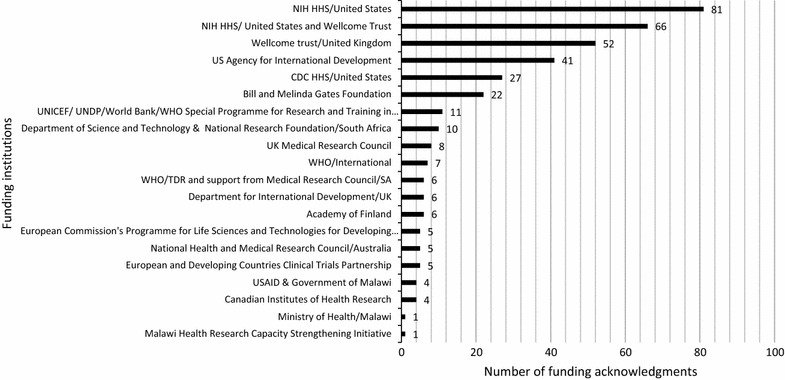
Malaria research funders in Malawi acknowledged in publications

Funding acknowledgements were assessed in the accessed full papers. One of the limitations was that only 457 (94.6%) full papers out of 483 publications included in the review could be retrieved. In addition, 410 (89.7%) out of 457 full papers acknowledged their source of funding (Fig. 2). There were several papers that acknowledged more than one funder and Fig. 8 only shows funding acknowledgments for a funder(s) in four (1%) or more publications. Similarly, details of approved studies from ECs were incomplete as only 37 (22.4%) out of 165 studies indicated their source of funding.

The National Institute of Health, Health and Human Sciences (NIH HHS) was the highest funding institution with 81 (20%) funding acknowledgements, followed by co-funding institutions of NIH HHS and the Wellcome Trust, UK, with 66 (16%) funding acknowledgements. Funding acknowledgements from Malawian institutions were explored and four (1%) publications were jointly funded by USAID and the Government of Malawi, while the Malawi Ministry of Health and Malawi Health Research Capacity Strengthening Initiative funded one (0.2%) research each. It is of interest to note that five out of these studies funded by the Malawian institutions and those with partners are mainly contextual studies that seek to understand the morbidity, epidemiology, and implementation of interventions.

**Fig. 9:**
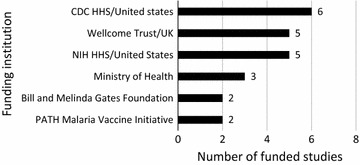
Malaria research funders in Malawi acknowledged in the approved studies

Thirty-seven (22.4%) of the ethically approved studies indicated their source of funding. Figure 9 shows analysis of approved studies from funding institutions that funded two or more studies (n = 23). The Centre for Disease Control Health and Human Services (CDC HHS) funded six studies while the Malawian Ministry of Health funded three studies.

**Fig. 10:**
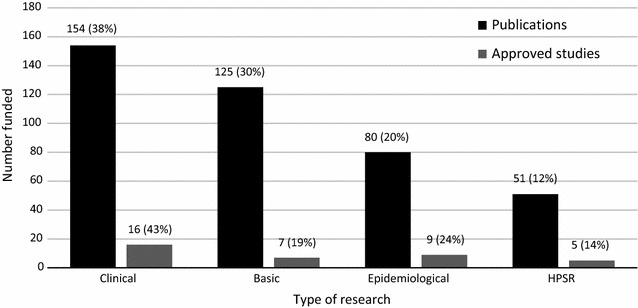
Most funded malaria research type in Malawi

The analysis showed that clinical research was highly funded, with 154 (38%) out of 410 publication acknowledgments and 16 (43%) of approved studies, while 51 (12%) of publications and five (14%) of approved studies in HPSR were the least to be funded as acknowledged (Fig. 10).

## Discussion

The review focused on assessing the type and amount of malaria research conducted in Malawi from 1984 to 2016 and its related source of funding. Bibliometric analysis was utilized to measure the number of publications from primary malaria research conducted in Malawi as a measure of scientific research activity. A total of 483 publications of primary malaria research originating from Malawi were online and 165 malaria approved studies from ECs. Malaria research activity in Malawi has steadily grown from two publications in 1984 to 51 in 2015 and 41 in 2016, and from one approved study in 2005 to 26 in 2016. This growth is linked to the research capacity in Malawi coinciding with the establishment of the COM in 1991 and its affiliates, such as the MAC, MLW and BMP. This has also shown that the majority of researchers are affiliated to these institutions as revealed in the assessment of first and senior/last authors. Other notable research institutions conducting malaria research in Malawi include the University of North Carolina (UNC) project, and the Ministry of Health. This rise in research activity contributed to Malawi being ranked eighth in the top African countries publishing malaria research between 1995 and 1997 [15]. Another important aspect revealed in this assessment was the level of collaboration. It is through collaboration that local capacity building can be strengthened although the level of involvement in the research is critical to ascertain this. The study has shown high collaboration, from publications, between one or more institutions in Malawi and one or more foreign institutions. It would have been ideal to establish the origins of authors and assess how many Malawian researchers were involved in the studies but ascertaining this based on names alone was a challenge (because last names may change through marriage, hence, being categorized differently) leading to underestimation or overestimation.

The type of research conducted in Malawi has shown that clinical and basic research are extensively conducted, with HPSR being the least supported. A similar review of research on infectious and non-infectious diseases in Malawi for the purposes of research gap analysis in the development of a national health research agenda revealed that clinical research was common while HPSR was the least [16]. The type of research reflects the research focus of COM and its affiliates in conducting clinical and basic research in malaria in pregnancy, severe malaria, and vector and/or agent dynamics. Similarly, these institutions will also acknowledge their major funders, for example, BMP attracts funding from NIH, USA, and MLW attracts funding from the Wellcome Trust, UK, which has also been identified to be the UK largest funder of infectious disease research to countries with colonial ties and Malawi is ranked as the fourth highest beneficiary [17]. This high reliance on external support for research funding is also reflected in the amount of funding from external support for malaria control [18].

The quality of these publications was not assessed due to the large volumes handled. However, the fact that they were published in reputable journals signifies that they underwent thorough peer review and were checked for quality. Malaria research from Malawi was mainly published in international journals since only three African journal were identified in this study and these included the *African Journal of Health Sciences*, *African Health Sciences*, and the *Malawi Medical Journal*. The major publisher of malaria research from Malawi is *Malaria Journal*, followed by the *American Journal of Tropical Medicine and Hygiene* and of special interest was the local *Malawi Medical Journal* indexed in Medline with 13 (2.7%) publications. This should be a platform to encourage local researchers to publish in local and regional journals as they are easily accessible to local policy makers and have been shown to influence policy change more than European or American journals [19]. Local academic institutions should put equal weight on the basis for promotion to publications in these journals as long as they are indexed in reliable databases [11].

Funding acknowledgements from publications show that NIH, USA topped the list, followed by joint funding by NIH and Wellcome Trust. Funding acknowledgements may indicate support but do not show the exact amount of funds put into the research. Some funders partially support or provide infrastructure which may not be acknowledged. Funders may influence recognition to be acknowledged and keep records of publications they fund [20]. All in all funders and policy makers are obliged to assess the quality and impact of their research investments and one of the approaches is to quantify the publication output, hence publishing should be a requirement for every funded research [21].

Of interest were the acknowledgements from Malawi, which showed joint funding by the USAID and the Government of Malawi in four publications, while the Ministry of Health and the Malawi Research Capacity Strengthening Initiative were acknowledged in one publication each. This shows high reliance on external funding and the challenge of local research funding availability. This can further be related to external support of clinical and basic research, which attracts more funding and is likely to be published [15]. The HPSR, which is the least supported in the publications, addresses issues that provide remedies to local health systems and likely influences practice and policy development as it is contextual [22]. This type of research needs support from the government if it aims at improving the health system and policy implementation. The challenge also remains in its dissemination by publication because of the difficulty to be accepted in international journals [15]. However, as more local and regional journals, such as the *Malawi Medical Journal*, are being indexed in the Medline, this type of research should be encouraged for publication, undergo peer review and increase visibility and readability. Policies based on non-peer-reviewed work usually raise concerns on the quality of evidence used, which may later have implications on quality of services [23]. Policy makers should be involved in the research process from the beginning and encourage collaboration with academicians, who should be responsible for publishing work as part of their promotion criteria.

As the HPSR is focused on improving the local health systems, the government should be responsible for providing funding for such research. Malawi is in the process of renewing the National Health Research Agenda, which outlines the country’s research needs. This is a critical step that can be the foundation of resource mobilization. Through its wider dissemination, external funding institutions can be compelled to align their research focus to local needs. In addition, the government should commit research funding as agreed in the Abuja Declaration, setting aside 2% of the health budget to research [24].

### Limitation(s) of the study

The online search was only limited to the Medline/PubMed database, hence publications from Journals not indexed in this database may have been missed. However, all efforts were made to search from reference sections of full articles that were accessed for potential articles missed out in the initial search. In addition due to time and staff capacity the broad term of ‘malaria’ was used in the search with the assumption of capturing all malaria-related terms. However, this may have missed out other potential publications with terms not associated to malaria.

## Conclusion

Viable malaria research has been conducted in Malawi since 1984 with clinical and basic research leading in both publications and funding. The major sources of funding for malaria research in Malawi come from NIH, USA, and the Wellcome Trust, UK, whose institutions, the BMP and MLW respectively, are affiliated to COM. The research focus of these institutions is reflected in the findings of this review, as clinical and basic research dominate in the fields of malaria in pregnancy, severe malaria and vector and/or agent dynamics. The least supported HPSR provides contextual evidence for the improvement of the health systems. As Malawi embarks on renewing the National Health and Research Agenda (NHRA), it is important that great attention is be placed on conducting HPSR, which will serve to understand the delivery of health services in Malawi and community dynamics in policy adoption during implementation. This, therefore, calls for government commitment to mobilize resources to support such research, which should also be encouraged for publication in local or regional journals indexed in major databases. Similarly, local malaria researchers should be aggressive in resource mobilization, such as grant applications, in order to conduct research that addresses local needs, as stipulated in the NHRA.

The quantity of publications, which may reflect the scientific research activity of a country, does not alone reveal the primary impact of research [11], i.e., improvement of public health. One immediate way of assessing this is to examine how development of health policies has been informed by local research.

## Abbreviations

BMP: Blantyre Malaria Project
CDC: Centre for Disease Control
CDC HHS: Centre for Disease Control Health and Human Services
COM: College of Medicine
COMREC: College of Medicine Research and Ethics Committee
CRH: Centre for Reproductive Health
ECs: Ethical Committees
HPSR: Health Policy and Systems Research
MAC: Malaria Alert Centre
MESH: Medical Subject Headings
MLW: Malawi-Liverpool Wellcome
NHSRC: National Health Sciences Research Committee
NIH HHS: National Institute of Health, Health and Human Sciences
NMCP: National Malaria Control Programme
OECD: Organization for Economic Co-operation and Development
UNC: University of North Carolina
USAID: US Agency for International Development
